# The Hospital Sunday Fund and Institution for the Incurables

**Published:** 1890-01-18

**Authors:** 


### Fallow Crops.

### THE HOSPITAL SUNDAY FUND AND INSTITUTIONS FOR THE INCURABLE.

The Royal Hospital for Incurables cannot reconcile itself to its exclusion from a share in the Metropolitan Hospital Sunday Fund. Therefore in the report now presented to their subscribers "the board renew their protest against what they feel to be the arbitrary exclusion of a hospital that undertakes the permanent care of cases which the general hospitals are powerless to relieve," and observe that "the Hospital Sunday Fund increases in volume annually and threatens to swamp the offerings to special hospitals whose operations do not commend themselves to its council."

Upon reading these comments the mind naturally rests for a moment upon the reflection—so trite in most people's experience—that it is very difficult to please everybody. While one set of non-contents is entering a protest of this description, there is another which puts so little value upon the privilege of participation that it would like, so it says, to see the fund abolished, for the altogether opposite reason that it is too insignificant to be of service, and supplies a very insufficient substitute for offerings which, without its meddling, would be forthcoming in fuller measure.

The exclusion of institutions for the incurable was decided upon at the outset, with the approval of the constituents. Therefore, the action of the council being strictly constitutional cannot, with any propriety of language, be accounted "arbitrary."

It is, however, open to the malcontents to move that this rule be modified or rescinded, and when this is formally done, the case against the Royal Hospital may be put thus:

The fund was originated in aid of institutions engaged in the medical and surgical treatment of disease, that is to say, hospitals and dispensaries. The Royal Hospital is an hospital only in name. To undertake "the permanent care" of the incurable is not the work of an hospital, and the difference is effectually marked (1) by the absence of a visiting staff of physicians and surgeons, and of all the manifold routine of hospital work, (2) by the method of admitting the inmates, which is by the votes of the whole body of subscribers.

The hospital proper is concerned with the malady; the home with the subject of the malady. Thus the main object and purpose of the former is to employ the utmost of scientific skill in the cure of sick people, and failing cure, to afford them such relief as may reconstitute them useful members of society. As secondary objects, there are the acquisition and diffusion of knowledge. The Hospital for Incurables deals with those whose maladies are beyond the reach of science, and requires from every applicant a medical certificate that he is an incurable. These are fundamental differences, and they are still further accentuated by the fact that by far the larger number of the former's beneficiaries receive nothing but a periodical gift of money.

Beyond all this, there is much which might be urged in opposition. The Sunday Fund was started in relief of the notorious and increasing poverty of our medical institutions, which are involved in a work incapable by its nature of being kept within definite dimensions, or made subordinate to its cost. An hospital is in the position of a firm which must honour at sight, by day or by night, Sundays and holidays, demands that have not been notified or provided for. The Royal Hospital, on the contrary, dispenses its benefits duly measured and weighed at fixed times, and demands for every admission granted, or pension bestowed, an equivalent in so many thousand votes, each representing some part of a subscription, while the friends of every candidate, if they be solicitous for his success, must bestir themselves not only to obtain votes but to collect subscriptions, and not infrequently, sooner than their candidate shall fail, they buy votes of the institution.

We are not now discussing the merits of the canvassing and voting system, nor are we in any way depreciating the value of the Royal Hospital in its own sphere of usefulness, but justice, no less than expediency, appears to require that the rule which excludes it from a share of the Hospital Sunday Fund shall be sustained. Agricola.

